# Anesthetic Management of a Surgical Patient with Chronic Renal Tubular Acidosis Complicated by Subclinical Hypothyroidism

**DOI:** 10.1155/2016/2434381

**Published:** 2016-08-28

**Authors:** Hiroe Yoshioka, Haruyuki Yamazaki, Rie Yasumura, Kosuke Wada, Yoshiro Kobayashi

**Affiliations:** Department of Anesthesiology, National Hospital Organization Tokyo Medical Center, 2-5-1 Higashigaoka, Meguro-ku, Tokyo 152-8902, Japan

## Abstract

A 53-year-old man with chronic renal tubular acidosis and subclinical hypothyroidism underwent lower leg amputation surgery under general anesthesia. Perioperative acid-base management in such patients poses many difficulties because both pathophysiologies have the potential to complicate the interpretation of capnometry and arterial blood gas analysis data; inappropriate correction of chronic metabolic acidosis may lead to postoperative respiratory deterioration. We discuss the management of perioperative acidosis in order to achieve successful weaning from mechanical ventilation and promise a complete recovery from anesthesia.

## 1. Introduction

Metabolic acidosis is categorized clinically as high or normal anion gap based on the presence or absence of unmeasured anions in serum. Renal tubular acidosis (RTA) is characterized by normal anion-gap metabolic acidosis, originating from excessive urinary loss of bicarbonate or defective urinary acidification [1]. Therefore, unlike high anion-gap acidoses (e.g., lactic acidosis or ketoacidosis), RTA must be treated with administration of sodium bicarbonate. However, perioperative acid-base management in such cases poses many difficulties because the total carbon dioxide (CO_2_) content in blood as well as actual blood pH must be fully taken into consideration for successful weaning from mechanical ventilation. In addition, thyroid function is associated with basal metabolic rate and CO_2_ production; thus, subclinical hypothyroidism presents specific challenges for anesthesiologists.

This case highlights the perioperative acid-base management of a patient who has suffered from untreated chronic RTA complicated by subclinical hypothyroidism while undergoing lower leg amputation surgery under general anesthesia. We report this case because the anesthetic management of similar cases has rarely been reported; in addition, there has been no prior report detailing the management of perioperative acidosis, which is the focal point of this case.

## 2. Case Presentation

A 53-year-old man (height: 167 cm; weight: 48 kg) who presented difficulty in walking due to severe pain in his lower leg was brought to our hospital by an ambulance. The patient had a prior history of chronic kidney disease, arteriosclerosis obliterans, and insulin-dependent diabetes mellitus. Physical and laboratory examination revealed ulcerative formations in the left lower leg and severe metabolic acidosis (Table 1). An intravenous subsequently oral administration of sodium bicarbonate resulted in marked improvement of the acidosis. Upon detailed examination, polyarteritis nodosa was strongly suspected as the etiology of the refractory ulcer in the lower leg; simultaneously, RTA type 4 was diagnosed based on a prior history of hyperkalemia and diabetes, accompanying a low serum aldosterone level.

For suspected pathophysiology, a 10 mg daily oral dose of prednisolone had been administered for about 2 months but his ulcerative lesions deteriorated; therefore, the patient was scheduled for lower leg amputation surgery about 3 months after admission.

Preoperative laboratory tests (Table 2) revealed impaired renal function and hyperkalemia, while those findings were compatible with RTA type 4. With regard to endocrine function, we suspected subclinical hypothyroidism due to reduced plasma triiodothyronine (T3) levels and elevated thyroid-stimulating hormone (TSH). Blood gas analysis showed severe metabolic acidosis accompanied by normal anion gap corrected for albumin and compensatory respiratory alkalosis (Table 1), because the oral sodium bicarbonate therapy had been discontinued 2 months before the surgery. Despite severe metabolic acidosis, the history and physical examination did not indicate any obvious evidence of hyperventilation. The patient had a body temperature of 36.4°C, blood pressure of 121/73 mmHg, a heart rate of 89 beats per minute (bpm), a respiratory rate of 10 breaths per minute, and an oxygen saturation level of 98% (room air); chest radiography and electrocardiogram examinations revealed no abnormalities.

General anesthesia was scheduled instead of regional anesthesia because of low platelet count. Oral prednisolone (10 mg/day) was continued until the day of the surgery. Preoperative values of blood pressure, heart rate, respiratory rate, and body temperature were almost within normal ranges: 142/91 mmHg, 84 bpm, 10 breaths per minute, and 35.8°C, respectively. Endotracheal intubation under general anesthesia was provided with intravenous induction of 50 *μ*g fentanyl, 70 mg propofol, and 40 mg rocuronium; and anesthesia was maintained by 1.2–1.8% sevoflurane with intermittent intravenous administration of fentanyl. The total amount of fentanyl was 250 *μ*g during operation. The end tidal CO_2_ (ETCO_2_) level of the patient was 22 mmHg immediately after intubation; then his minute volume was set at 3 L/min. However, the blood gas analysis revealed that the arterial carbon dioxide partial pressure (PaCO_2_) of the patient was within the normal range (Table 1). The frequency of ventilation was subsequently adjusted to and maintained at a rate of 3–3.5 L/min, in order to ensure that the PaCO_2_ remained within the normal range. During this time, ETCO_2_ levels were 22–29 mmHg.

The total time of surgery was 1 h and 48 min; 1250 mL of crystalloid solution (normal saline and hypotonic solution which contains no potassium), 280 mL of red blood cell transfusion, and 100 mL of 8.4% sodium bicarbonate were administered during the surgery. The total blood loss and urine volume were determined to be 170 mL and 150 mL, respectively. Although the patient continued to exhibit mild signs of metabolic acidosis upon completion of surgery (Table 1), this did not appear to impact his circulatory condition. A chest radiograph taken upon completion of surgery revealed a small amount of bilateral pleural effusion. The patient was given a muscle relaxant antagonist (200 mg of sugammadex) after awakening and was extubated after a spontaneous breathing trial. There was no appreciable event during the surgery, except a red blood cell transfusion. No subsequent problems related to hyperventilation or apnea were observed, and the patient was sent back to his room without incident.

## 3. Discussion 

RTA is classified into three types based on pathophysiology. Among them, RTA type 4 is characterized by distal tubular aldosterone resistance or aldosterone deficiency, resulting in hyperkalemia and onset of metabolic acidosis [1]. RTA type 4 is usually asymptomatic with only mild acidosis but can be occasionally accompanied by life-threatening electrolyte disturbances and severe decrease in bicarbonate concentration. In addition, its clinical course tends to be prolonged and thus complicated as with this case. Therefore, rather than actual blood pH, time-dependent changes in contained CO_2_ in the whole body should be taken into consideration for successful weaning from mechanical ventilation when perioperative corrective treatment is performed.

Literature reviews of anesthetic management of patients with severe acute metabolic acidosis have focused on the maintenance of adequate blood pressure and tissue perfusion. However, in chronic cases, anesthesiologists should give attention to intraoperative acid-base status in order to ensure adequate spontaneous breathing immediately after surgery.

In general, respiratory drive is stimulated by low pH, particularly in patients with chronic metabolic acidosis. Therefore, any arterial pH, which is higher than preoperative value, can be associated with a higher risk for postoperative hypoventilation to various degrees, even if normal acid-base balance without hypercapnia has been achieved at the time of weaning from mechanical ventilation. Rapid or excessive correction of chronic metabolic acidosis during surgery leads to either life-threatening hypoventilation or sudden onset of apnea [2].

With regard to correction of acidosis, there is no conclusive evidence to support bicarbonate administration to surgical patients with chronic metabolic acidosis. In contrast, patients of RTA type 4 need administration of bicarbonate at a daily rate of 1.5–2 mmol/kg [1]; nevertheless, the dose and rate of perioperative bicarbonate administration remain controversial in particular cases.

In general, severe metabolic acidosis with arterial pH < 7.2 is associated with higher mortality in critically ill patients [3]; therefore, we adjusted the ventilator settings and bicarbonate administration to maintain arterial pH at a value simultaneously less than the preoperative value and higher than 7.2 along with normocapnia.

On the other hand, the clinical picture of this case was remarkable in the light of perioperative PaCO_2_ level when compared to that of most patients with severe metabolic acidosis.

Firstly, although the preoperative PaCO_2_ of the patient was extremely low (27.7 mmHg), which seems to be respiratory overcompensation, no obvious signs of deep breathing or hyperventilation were observed. Secondly, despite the adjustment of the minute volume in order to bring the PaCO_2_ level within the normal range, low minute volume was required for this patient. For comparison, the minute volume and the PaCO_2_ level were assessed in other surgical patients without metabolic acid-base disorders (Table 3). The minute volume and the PaCO_2_ level exhibited by the patient in this case were lower than the group average. Based on these observations, we speculated that any underlying pathophysiologies might lead to a decrease in PaCO_2_ without an increase in alveolar ventilation.

Specifically, the body equilibrium of RTA patients is known to shift to the left as follows: H^+^ + HCO_3_^−^ *⇄*  H_2_CO_3_  ⇄  H_2_O + CO_2_; as such, if the bicarbonate level is not replenished, the internal CO_2_ level will be depleted, resulting in a decline in PaCO_2_.

As another speculation, subclinical hypothyroidism may possibly lead to decreased CO_2_ production in the body because decreased thyroid function is associated with diminished basal metabolic rate, thus resulting in reduced tissue CO_2_ production [4]; similarly, subclinical hypothyroidism is reported to induce a decrease in resting energy expenditure [5].

Definitely, subclinical hypothyroidism and RTA type 4 have the potential to complicate the reading and interpretation of capnometry and PaCO_2_ measurements.

In order to avoid postoperative respiratory deterioration in patients with chronic metabolic acidosis, anesthesiologists should give basic consideration to the previously documented pitfalls: type of anesthesia, dose of opioids used in combination, methods of postoperative pain management, and body temperature of the patient. Besides, perioperative acid-base management on the basis of the abovementioned goal of pH and PaCO_2_ will probably determine success of weaning from mechanical ventilation and promise a complete recovery from anesthesia in patients with RTA complicated by subclinical hypothyroidism. Given that hemodynamic stability has already been achieved in patients suffering from long-term preoperative exposure to RTA with low serum bicarbonate concentration, the main anesthetic concerns should include the dose and rate of bicarbonate administration tailored according to the patient's usual acid-base balance and preoperative respiratory status: PaCO_2_ levels, the minute volume, and the presence of signs of deep breathing or hyperventilation.

## Figures and Tables

**Table 1:** Perioperative blood gas analysis.

	On admission	Preoperative	Immediately after intubation	Postoperative
pH	7.031	7.345	7.286	7.345
PaCO_2_ (mmHg)	13.6	27.7	34.4	37.1
HCO_3_^−^ (mmol/L)	3.5	14.8	16.0	19.8
BE (mmol/L)	−25.3	−10.0	−9.7	−5.4
AnGap (mmol/L)	18.1	3.6	4.8	2.7
Lactate (mg/dL)	15.4	17.4	—	—

AnGap: anion gap; BE: base excess; HCO_3_^−^: bicarbonate; PaCO_2_: arterial carbon dioxide partial pressure.

**Table 2:** Preoperative laboratory test values.

	Test value (normal range)
HGB (g/dL)	7.1 (13.5–17.0)
PLT (×10^9^/L)	59 (150–350)
BUN (mg/dL)	32.7 (8.0–22.0)
Cre (mg/dL)	2.6 (0.6–1.1)
TP (g/dL)	4.6 (6.3–8.2)
Alb (g/dL)	2.1 (3.5–5.2)
Na (Mmol/L)	128 (138–146)
K (Mmol/L)	5.3 (3.6–4.9)
Cl (Mmol/L)	106 (99–109)
PRA (ng/mL/h)	0.3 (0.2–2.7)
PAC (pg/mL)	13 (36–240)
TSH (U/dL)	6.17 (0.3–4.5)
T3 (pg/mL)	1.2 (2.0–4.5)
T4 (ng/dL)	1.1 (0.7–1.8)

Alb: albumin; BUN: blood urea nitrogen; Cl: chloride; Cre: creatinine; HGB: hemoglobin; K: potassium; Na: sodium; PAC: plasma aldosterone concentration; PRA: plasma renin activity; TP: total protein; TSH: thyroid-stimulating hormone; T3: triiodothyronine; T4: thyroxine.

**Table 3:** Minute volume and PaCO_2_ levels of 29 male patients (aged 48–90) subjected to nonlaparoscopic surgery.

	Mean (SD)
Age (years)	74.0 (11.2)
Height (cm)	165.0 (5.2)
Weight (kg)	48.7 (2.2)
Minute volume (L/min)	4.8 (1.0)
PaCO_2_ (mmHg)	39.1 (5.0)

PaCO_2_: arterial carbon dioxide partial pressure.
